# One-year hospital readmission for recurrent sepsis: associated risk factors and impact on 1-year mortality—a French nationwide study

**DOI:** 10.1186/s13054-022-04212-9

**Published:** 2022-11-29

**Authors:** Fanny Pandolfi, Christian Brun-Buisson, Didier Guillemot, Laurence Watier

**Affiliations:** 1 Epidemiology and Modeling of Bacterial Evasion to Antibacterials Unit (EMEA), Institut Pasteur, Université Paris Cité, Paris, France; 2 Centre de Recherche en Epidémiologie et Santé des Populations (CESP), Institut National de la Santé et de la Recherche Médicale (INSERM), Université de Versailles Saint Quentin-en-Yvelines/Université Paris Saclay, Paris, France; 3 AP-HP, Paris Saclay, Public Health, Medical Information, Clinical Research, Le Kremlin-Bicêtre, France

**Keywords:** Hospital readmission, Infection, Intensive care unit, Long-term care, Septic shock, Sepsis

## Abstract

**Background:**

Sepsis is a complex health condition, leading to long-term morbidity and mortality. Understanding the risk factors for recurrent sepsis, as well as its impact on mid- and long-term mortality among other risk factors, is essential to improve patient survival.

**Methods:**

A risk factor analysis, based on French nationwide medico-administrative data, was conducted on a cohort of patients above 15 years old, hospitalized with an incident sepsis in metropolitan France between 1st January 2018 and 31st December 2018 and who survived their index hospitalization. Two main analyses, focusing on outcomes occurring 1-year post-discharge, were conducted: a first one to assess risk factors for recurrent sepsis and a second to assess risk factors for mortality.

**Results:**

Of the 178017 patients surviving an incident sepsis episode in 2018 and included in this study, 22.3% died during the 1-year period from discharge and 73.8% had at least one hospital readmission in acute care, among which 18.1% were associated with recurrent sepsis. Patients aged between 56 and 75, patients with cancer and renal disease, with a long index hospital stay or with mediastinal or cardiac infection had the highest odds of recurrent sepsis. One-year mortality was higher for patients with hospital readmission for recurrent sepsis (aOR 2.93; 99% CI 2.78–3.09). Among all comorbidities, patients with cancer (aOR 4.35; 99% CI 4.19–4.52) and dementia (aOR 2.02; 99% CI 1.90–2.15) had the highest odds of 1-year mortality.

**Conclusion:**

Hospital readmission for recurrent sepsis is one of the most important risk factors for 1-year mortality of septic patients, along with age and comorbidities. Our study suggests that recurrent sepsis, as well as modifiable or non-modifiable other risk factors identified, should be considered in order to improve patient care pathway and survival.

**Supplementary Information:**

The online version contains supplementary material available at 10.1186/s13054-022-04212-9.

## Background

Sepsis is a complex health condition, leading to long-term morbidity, high level of mortality and substantial economic burden for the health care system [1–4]. Severe sequelae resulting from sepsis episodes can alter patients health condition, increase the risk of hospital readmission and also reduce their life expectancy [3, 5]. Sepsis is associated with a high risk of hospital readmission and recurrent sepsis is also the most common cause of hospital readmission in previous studies [1, 6, 7]. However, other causes are also reported [2, 8, 9]. Hospital readmission can be influenced by several factors such as age, specific health conditions, social background, comorbidities and acute injuries [1, 6, 10–12] but some of these risk factors like age or important comorbidities as well as ICU and mechanical ventilation were not significantly different between septic patients with and without recurrent sepsis [7]. More generally, risk factors for recurrent sepsis are seldomly studied. The risk of readmission is usually higher in the first weeks after the index hospitalization, but it could eventually occur after a longer period [6, 7]. Several studies focussed on 30 or 90-day readmission after the index admission for sepsis but few studies focussed specifically on hospital readmission for recurrent sepsis over a longer period and its associated risk factors [4, 7, 13].

The mortality rate for sepsis is relatively high. Studies are hardly comparable because of differences in the study population, the methods of selection for sepsis cases and changes in sepsis definition across the study periods. In recent studies, in-hospital mortality rate varied from 11.8 to 35.8%, 30-day mortality between 8.3 and 26.5% [5, 14–19] and 1-year mortality from 12.5 to almost 50% [5, 20]. The identified risk factors for mid- to short-term mortality include age, comorbidities, multiple organ failure and septic shock [14, 15, 21–24]. Short-term mortality is usually higher in the elderly. However, short-term assessment underestimate the possible sequelae of sepsis which may lead to possible hospital readmission, or other risk factors for long-term mortality in sepsis survivors [6, 7, 22, 25]. DeMerle and al. showed that 90-day mortality was higher for septic patients readmitted for sepsis [7]. However, the risk factors for long-term mortality of sepsis survivors, and in particular the association between hospital readmission for recurrent sepsis and long-term mortality, have been scarcely studied [6, 26]. To the best of our knowledge, no nationwide study was recently published in France on this specific topic.

The aim of this nationwide study was to assess the risk factors for 1-year hospital readmission for recurrent sepsis, as well as the possible impact of recurrent sepsis and other risk factors as age and comorbidities on 1-year mortality in sepsis survivors.

## Methods

### Data source, definitions, and study population

The study consisted of a secondary data analysis of a cohort of all patients with bacterial infections and registered in the anonymized French National Hospital Discharge Database (Programme de Médicalisation des Systèmes d'Information: PMSI) and outpatient health care consumption (Données de Consommation Inter-Régimes: DCIR) issued from the French health care database (Système National des Données de Santé: SNDS) (see Additional file 1: eMethods) [27, 28]. Therefore, only cases of sepsis of presumed bacterial etiology (referred to herein as sepsis) were considered.

Targeting to select sepsis related hospital stays, sepsis was identified as the combination of explicit sepsis and implicit sepsis [18, 29, 30]. The selection in the French medico-administrative database was based on a list of ICD-10 codes, organ dysfunction and ICU admission (see Additional file 1: eMethods and eTable 1). More details about the selection can be found in our previous study [27]. Only hospital stays in an acute-care facility (MSO: medicine surgery and obstetrics) were considered. Stays shorter than one day where the patient did not die were excluded.

The study population included all patients above 15 years old, hospitalized with an incident sepsis (index hospitalization) in metropolitan France between 1st January 2018 and 31st December 2018 and who survived their index hospitalization. Patients with an incident sepsis were identified as patients who did not experience a sepsis-related hospitalization during the previous 12 months of their first sepsis-related hospitalization in 2018. Recurrent sepsis was defined by at least one sepsis-related hospital readmission during the 12-month period following hospital discharge of the index sepsis hospitalization; 1-year mortality was defined as patient death during the same period.

Patient, infection, and hospitalization characteristics such as sex, age, Charlson index and detailed comorbidities, infection site, admission source, hospital discharge, length of stay, day of death, admission to ICU and septic shock were recorded (see Additional file 1: eTable2). One-year hospital readmissions in MSO for sepsis or other diagnoses were recorded.

### Statistical analyses

Patients were categorized and described according to 1-year hospital readmissions for recurrent sepsis as well as 1-year mortality. Hospital stays and site of infections were also described for these categories as well as acute cardiac events. The mortality of sepsis survivors was estimated at 30 days, 90 days, 6 months and 1 year following discharge of the index-hospitalization and stratified according to 1-year hospital readmission for recurrent sepsis.

Firstly, patients, infection and index hospital stay characteristics were described according to the variables of interest (re-hospitalized for recurrent sepsis vs not re-hospitalized or re-hospitalized for other causes; 1-year mortality vs alive). Frequencies (percentages) were reported for discrete variables and median and interquartile range (IQR) for quantitative ones. As the data cover the national population, no confidence intervals were calculated for percentages [31, 32].

Secondly, two main analyses were conducted on the selected survivor population. The first analysis aimed at identifying risk factors for re-hospitalization with recurrent sepsis during the 1-year period from discharge of the index hospitalization. In order to improve the robustness of our results, this analysis was repeated on two subpopulations of sepsis survivors: only those experiencing hospital readmission (for sepsis or any other reason), and on sepsis survivors with explicit sepsis only. The second analysis aimed at identifying risk factors for 1-year mortality post-discharge. This analysis was also repeated on sepsis survivors with explicit sepsis only. Logistic regressions were used to identify risk factors associated with the two studied outcomes: recurrent sepsis or 1-year mortality. For the latter, a Cox regression was also used to assess hazard ratio for 1-year mortality. One-year Kaplan–Meier crude survival curve was also produced.

We conducted a univariate analysis including potential confounders and risk factors followed by a multivariate analysis. Risk factors considered are listed in Tables 1 and 2. As ICU admission and septic shock were not independent, a 4-classes variable was constructed: the absence of both, the presence of ICU admission only, the presence of septic shock only and the presence of both (see Additional file 1: eTable2). Since individual comorbidities were considered, the Charlson index, a score which combines comorbidities, was not included in the logistic regressions. The variables for which the 99% CI of the crude odd ratio or hazard ratio (cOR/cHR) did not contain 1 and with a global *P*-value < 0.01 were included in the multivariate analysis. As the data cover the national population, a conservative 2-tailed *P*-value < 0.01 in the multivariable model defined significance [31, 32].

**Table 1:** Risk factors, recorded during the index hospitalization, for 1-year hospital readmission for recurrent sepsis for 178,017 adult patients hospitalized with sepsis in metropolitan France in 2018: univariate and multivariate logistic regressions

	cOR^a^ (99% CI)	*P* value	aOR^a^ (99% CI)	*P* value
Patient characteristics				
Sex (ref = men)	0.76 (0.73–0.78)	< 0.001	0.85 (0.82–0,89)	< 0.0001
Age (ref = 16–30)		< 0.001		< 0.001
31–45	1.37 (1.19–1.58)		1.21 (1.04–1.39)	
46–55	1.80 (1.58–2.06)		1.35 (1.18–1.54)	
56–65	2.17 (1.92–2.46)		1.51 (1.33–1.72)	
66–75	2.28 (2.01–2.57)		1.57 (1.38–1.78)	
76–85	1.87 (1.66–2.12)		1.40 (1.23–1.59)	
> 85	1.37 (1.20–1.55)		1.14 (1.00–1.31)	
Heart failure (ref = no)	1.09 (1.04–1.14)	< 0.001	1.14 (1.09–1.19)	< 0.001
Dementia (ref = no)	0.68 (0.62–0.75)	< 0.001	0.82 (0.74–0.90)	< 0.001
Chronic pulmonary disease (ref = no)	1.08 (1.02–1.14)	< 0.001	1.08 (1.02–1.14)	0,001
Rheumatologic disease (ref = no)	1.21 (1.04–1.41)	0.001	1.33 (1.14–1.55)	< 0.001
Liver disease (ref = no)	1.45 (1.35–1.56)	< 0.001	1.33 (1.23–1.43)	< 0.001
Diabetes with chronic complications (ref = no)	1.49 (1.39–1.59)	< 0.001	1.29 (1.20–1.39)	< 0.001
Paraplegia and hemiplegia^b^ (ref = no)	1.00 (0.92–1.08)	0.901	–	
Renal disease (ref = no)	1.54 (1.47–1.62)	< 0.001	1.55 (1.47–1.63)	< 0.001
Cancer (ref = no)	2.14 (2.06–2.23)	< 0.001	1.98 (1.90–2.07)	< 0.001
AIDS HIV (ref = no)	1.36 (1.06–1.74)	0.001	1.30 (1.01–1.67)	0.008
Hospital stay characteristics				
Hospital discharge (ref = Home)		< 0.001		< 0.001
Acute care	1.02 (0.97–1.07)		1.04 (0.99–1.10)	
Home care	1.47 (1.28–1.70)		1.06 (0.92–1.23)	
Long-term care	0.91 (0.86–0.95)		0.86 (0.82–0.91)	
Length of stay (in days) (ref ≤ 7)		< 0.001		< 0.001
7–30	1.26 (1.19–1.34)		1.16 (1.09–1.23)	
31–90	1.71 (1.60–1.83)		1.48 (1.37–1.59)	
> 90	2.11 (1.84–2.42)		1.98 (1.71–2.29)	
ICU admission and septic shock (ref = absence of both)			< 0.001 < 0.001	
No ICU admission and septic shock	0.82 (0.71–0.96)		0.91 (0.78–1.06)	
ICU admission and septic shock	1.07 (1.01–1.12)		0.97 (0.92–1.03)	
ICU admission and no septic shock	0.87 (0.84–0.91)		0.82 (0.79–0.86)	
Infection characteristics				
Site (ref = Urinary and genital tracts)		< 0.001		< 0.001
Gastrointestinal and abdomen	1.25 (1.15–1.36)		1.16 (1.06–1.26)	
Primary bacteraemia	1.49 (1.40–1.58)		1.28 (1.20–1.36)	
Bones and joints	1.14 (0.99–1.30)		(0.94–1.24)	
Heart and mediastinum	1.92 (1.76–2.09)		1.47 (1.35–1.61)	
Multiple	1.33 (1.25–1.41)		1.09 (1.03–1.17)	
Material device	1.35 (1.22–1.49)		1.06 (0.96–1.17)	
Lower respiratory tract	0.94 (0.89–1.01)		0.92 (0.86–0.99)	
Skin and soft tissues	1.37 (1.25–1.50)		1.23 (1.12–1.35)	
Others	0.47 (0.40–0.55)		0.54 (0.46–0.64)	

^a^cOR, Crude odds ratio; aOR, Adjusted odds ratio

^b^Variables excluded from multivariate analysis

**Table 2:** Risk factors for 1-year mortality of the 178,017 adult patients surviving a first episode of sepsis in metropolitan France in 2018: univariate and multivariate logistic regression

	cOR^a^ (99% CI)	*P* value	aOR^a^ (99% CI)	*P* value
Patient characteristics				
Sex (ref = men)	0.93 (0.90–0.95)	< 0.001	0.97 (0.94–1.01)	0.034
Age (ref = 16–30)		< 0.001		< 0.001
31–45	2.20 (1.83–2.65)		1.90 (1.57–2.30)	
46–55	4.13 (3.47–4.91)		2.84 (2.38–3.40)	
56–65	5.76 (4.87–6.81)		3.49 (2.93–4.14)	
66–75	7.12 (6.04–8.40)		4.33 (3.65–5.14)	
76–85	9.06 (7.68–10.69)		6.40 (5.39–7.59)	
> 85	14.01 (11.87–16.54)		11.33 (9.54–13.47)	
Heart failure (ref = no)	1.38 (1.34–1.43)	< 0.001	1.32 (1.27–1.38)	< 0.001
Dementia (ref = no)	2.42 (2.28–2.56)	< 0.001	2.02 (1.90–2.15)	< 0.001
Chronic pulmonary disease (ref = no)	1.11 (1.06–1.16)	< 0.001	1.18 (1.12–1.24)	< 0.001
Rheumathologic disease^b^ (ref = no)	0.99 (0.87–1.13)	0.908	─	
Liver disease (ref = no)	1.15 (1.07–1.22)	< 0.001	1.50 (1.40–1.61)	< 0.001
Diabetes with chronic complications(ref = no)	1.07 (1.01–1.14)	0.003	1.06 (0.99–1.14)	0.024
Paraplegia and hemiplegia^b^ (ref = no)	1.06 (1.00–1.13)	0.018	─	
Renal disease (ref = no)	1.46 (1.40–1.52)	< 0.001	1.29 (1.24–1.36)	< .0001
Cancer (ref = no)	3.71 (3.59–3.83)	< 0.001	4.35 (4.19–4.52)	< .0001
AIDS HIV (ref = no)	0.58 (0.45–0.76)	< 0.001	0.90 (0.68–1.19)	0.316
Hospital stay characteristics				
Hospital readmission^c^ (ref = no hospital readmission)		< 0.001		< 0.001
Recurrent sepsis	3.48 (3.32–3.64)		2.93 (2.78–3.09)	
Other acute care	1.41 (1.36–1.47)		1.25 (1.20–1.30)	
ICU admission and septic shock (ref = absence of both)		< 0.001		< 0.001
No ICU admission and septic shock	1.91 (1.72–2.11)		1.82 (1.63–2.04)	
ICU admission and septic shock	0.68 (0.65–0.71)		0.83 (0.78–0.87)	
ICU admission and no septic shock	0.62 (0.60–0.64)		0.73 (0.70–0.76)	
Infection characteristics				
Site (ref = Urinary and genital tracts)		< 0.001		< 0.001
Gastrointestinal and abdomen	0.91 (0.85–0.98)		0.98 (0.90–1.06)	
Primary bacteremia	1.50 (1.43–1.58)		1.39 (1.31–1.47)	
Bones and joints	0.65 (0.57–0.73)		0.87 (0.76–0.99)	
Heart and mediastinum	1.48 (1.37–1.60)		1.39 (1.28–1.51)	
Multiple	1.32 (1.26–1.38)		1.39 (1.32–1.46)	
Material device	1.26 (1.16–1.37)		1.20 (1.10–1.32)	
Lower respiratory tract	1.07 (1.02–1.13)		1.38 (1.30–1.45)	
Skin and soft tissues	1.67 (1.55–1.79)		1.72 (1.59–1.86)	
Others	0.42 (0.37–0.48)		0.94 (0.82–1.09)	

^a^cOR, Crude odds ratio; aOR, Adjusted odds ratio

^b^Variables excluded from multivariate analysis

^c^During the 1-year period following hospital discharge of the index sepsis-related hospitalization

## Results

### Selection of the study population

**Fig. 1:**
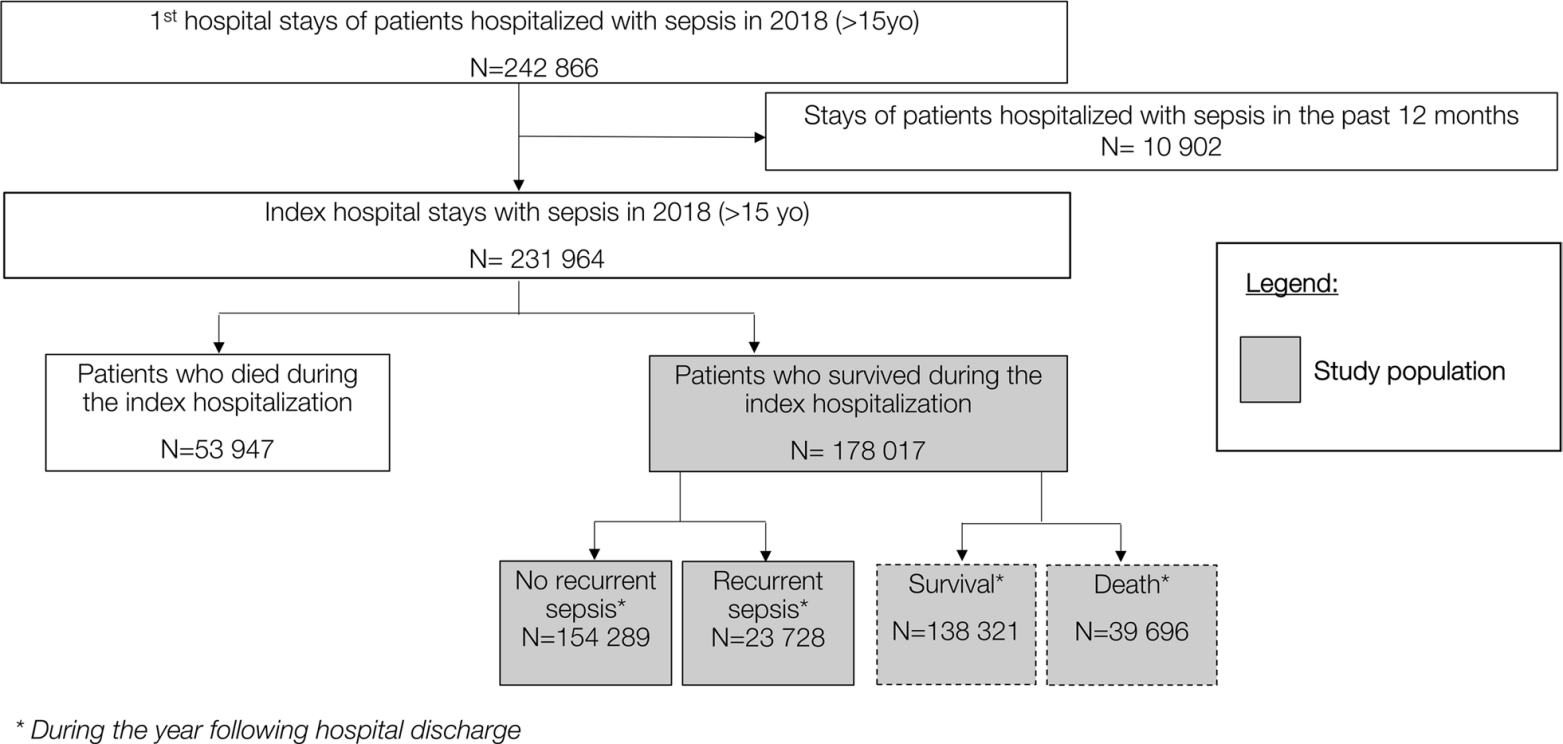
Flowchart of patient selection

In total, 231,934 patients were admitted with incident sepsis in 2018 of which 23.3% died during the index admission (Fig. 1). Of the 178,017 patients (140,658 with explicit sepsis and 37,359 with implicit sepsis) who survived the index hospitalization, 131,364 (73.8%) had at least one hospital readmission in MSO (all causes) in the year following hospital discharge, among which 23,728 (18.1%) were associated with recurrent sepsis.

### Recurrent sepsis during the 1-year period following hospital discharge

#### Description

Patients, infections, and hospitalization characteristics recorded during the index hospitalization, according to 1-year hospital readmission for recurrent sepsis are reported in Table 3. As compared with those having no recurrence, patients with recurrent sepsis were more often men (62.6% vs 55.8%) and had more often comorbidities, especially cancer (35.7% vs 20.6%). Regarding infection sites, recurrent sepsis was more often associated with primary bacteremia (19.6% vs 15.9%), multiple sites infection (23.9% vs 21.7%) and less frequently with urinary or genital tract infections (15.2% vs 18.3%). One-year mortality was also much higher for these patients (39.8% vs 19.6%). For patients who experienced recurrent sepsis the median (range) number of recurrences in the following year was 2 (2–14). The median number of days and IQR between the index hospitalization and the first recurrence was 76 (25–178) days.

**Table 3:** Comorbidities, demographic, hospital stay and infection characteristics of the 178,017 survivors of the index hospitalization according to 1-year hospital readmission for recurrent sepsis, metropolitan France 2018

	No hospital readmission for recurrent sepsis		1-year hospital readmission for recurrent sepsis	
	(*N* = 154,289)		(*N* = 23,728)	
	*N*	%	*N*	%
Patient characteristics^a^				
Sex				
Men	86,071	55.8	14,843	62.6
Women	68,218	44.2	8885	37.5
Age, median (IQR)	71 (59–82)		70 (61–80)	
16–30	6274	4.1	518	2.2
31–45	10,357	6.7	1172	4.9
46–55	14,277	9.3	2125	9.0
56–65	26,220	17.0	4702	19.8
66–75	36,462	23.6	6850	28.9
76–85	36,097	23.4	5586	23.5
> 85	24,602	16.0	2775	11.7
Charlson^a^, median (IQR)	2 (0–3)		2 (1–4)	
0	59,563	39.5	5550	23.4
1–2	53,588	35.5	9604	40.5
3–4	20,211	13.4	4163	17.5
> = 5	17,583	11.7	4411	18.6
Comorbidities				
Heart failure	31,468	20.4	5183	21.8
Dementia	8408	5.5	896	3.8
Chronic pulmonary disease	17,421	11.3	2871	12.1
Rheumatologic disease	1896	1.2	352	1.5
Liver disease	7521	4.9	1640	6.9
Diabetes with chronic complications	8708	5.6	1939	8.2
Paraplegia and hemiplegia	8373	5.4	1283	5.4
Renal disease	18,457	12.0	4113	17.3
Cancer	31,718	20.6	8468	35.7
AIDS HIV	638	0.4	133	0.6
Acute cardiac disease^a^				
During the index hospitalization	29,157	18.9	4776	20.1
In the following 12 months^b^	21,378 (11,782)	19.9(11.0)	7795 (4729)	32.9 (19.9)
Hospital stay characteristics				
Admission source^c^				
Acute care	13,286	8.6	2207	9.3
Home	138,447	89.7	21,090	88.9
Home care	258	0.2	68	0.3
Long-term care	2298	1.5	363	1.5
Hospital discharge^c^				
Acute care	23,825	15.4	3773	15.9
Home	98,753	64.0	15,340	64.7
Home care	1807	1.2	413	1.7
Long-term care	29,904	19.4	4202	17.7
Length of stay, days^c^				
< 7	22,086	14.3	2587	10.9
7–30	103,859	67.3	15,353	64.7
31–90	26,219	17.0	5263	22.2
> 90	2125	1.4	525	2.2
ICU admission and septic shock^c^				
No ICU admission and septic shock	2443	1.6	324	1.4
No ICU admission and no septic shock	69,785	45.2	11,241	47.4
ICU admission and septic shock	20,439	13.3	3508	14.8
ICU admission and no septic shock	61,622	39.9	8655	36.5
Number of recurrences				
0	154,289	100	–	–
1	–	–	18,519	78.0
2	–	–	3764	15.9
> 2	–	–	1445	6.1
Emergency admission^c^				
Yes	88,469	57.3	12,154	51.2^d^
No	65,820	42.7	11,574	48.8
Infection characteristics^c^				
Site of infection				
Gastrointestinal and abdomen	9769	6.3	1558	6.6
Primary bacteremia	24,584	15.9	4653	19.6
Bones and joints	3392	2.2	490	2.1
Heart and mediastinum	6484	4.2	1580	6.7
Multiple	33,527	21.7	5670	23.9
Medical device	5680	3.7	977	4.1
Lower respiratory tract	30,703	19.9	3684	15.5
Skin and soft tissues	7067	4.6	1233	5.2
Urinary and genital tracts	28,255	18.3	3595	15.2
Others	4828	3.1	288	1.2
1-year mortality				
Yes	30,249	19.6	9447	39.8
No	124,040	80.4	14,281	60.2

^a^ICD-10 codes: I20-I24, I50 or I64 as primary diagnosis, related diagnosis or significant associated diagnosis during hospitalization in acute care

^b^Acute cardiac disease in the following 12 months (acute cardiac disease in the following 12 months without previous onset during the index stay)

^c^During index hospitalization

^d^46.3% had emergency admission for the first recurrent sepsis after index hospitalization

#### Risk factors analysis

Among potential confounders or risk factors for recurrent sepsis, only one comorbidity (paraplegia and hemiplegia) was excluded from the multivariate analysis (Table 1). After adjustment, women had a lower aOR (adjusted Odds ratio) of hospital readmission for recurrent sepsis than men, and those aged between 56 and 75 yrs had the highest aOR compared to younger patients. Among all comorbidities, cancer (aOR 1.98; 99% CI 1.90–2.07) and renal disease (aOR 1.55; 99% CI 1.47–1.63) were associated with the highest aOR while dementia had an aOR lower than 1 (aOR 0.82; 99% CI 0.74–0.90). Compared to home discharge, the risk of hospital readmission for recurrent sepsis was lower for patients discharged to long-term care (aOR 0.89; 99% CI 0.84–0.95), whereas the aOR of hospital readmission was almost twice higher for a length of stay of the index admission > 90 days compared to one < 7 days. Hospital readmission for recurrent sepsis was lower with ICU admission, only for patients without septic shock (aOR 0.82; 99% CI 0.79–0.86). Compared to urinary and genital tracts infection, heart and mediastinum infection had the highest aOR (aOR 1.47; 99% CI 1.35–1.61). The directionality of the risk factors was similar when the analysis was conducted on the subgroup of patients with explicit sepsis only (see Additional file 1: eTable 3). However, when sepsis-related readmissions were compared to hospital readmission for other causes, discharge to acute care was identified as a protective factor for sepsis-related hospital readmission (aOR 0.83; 99% CI 0.79–0.88). In the latter analysis, the length of stay and some major comorbidities (cancer, renal and liver diseases, diabetes, AIDS) remained relevant risk factors for sepsis-related hospital readmission (see Additional file 1: eTable 4).

### Mortality during the 1-year period following hospital discharge

#### Description

Among patients who survived to their incident sepsis episode, 39,696 (22.3%) died in the year following hospital discharge of the index hospitalization. One-year mortality was much higher among patients with recurrent sepsis compared to patients which did not experience recurrent sepsis in the following year. Those who had died at 1-year were more often elderly (> 75 years), with a higher Charlson index and more often comorbidities, especially cancer (42.8% vs 16.8% among 1-year survivors). They also had more often bacteremia or multiple infections and less often urinary/genital tracts infection and a higher proportion of septic shock without ICU admission (Table 4, see Additional file 1: eTable 5).

**Table 4:** 30-day, 90-day, 6-month and 1-year mortality of adult patients surviving a first episode of sepsis in metropolitan France in 2018 according to hospital readmission for recurrent sepsis

Variables	Mortality (%)			
	30-days	90-days	6-month	1-year
Patients without recurrent sepsis	6.5	11.5	15.3	19.6
Patients with recurrent sepsis	3.9	13.7	24.5	39.8
All patients	6.2	11.8	16.5	22.3

#### Risk factors analysis

Among potential confounders or risk factors for 1-year mortality, two comorbidities (Rheumatologic disease and paraplegia/hemiplegia) were excluded from the multivariate analysis. Women had a lower aOR of 1-year mortality than men. As expected, mortality tended to increase with age, with the oldest age category (> 85 years) having the greatest odds of 1-year mortality (aOR 11.33; 99% CI 9.54–13.47). Among all comorbidities, cancer (aOR 4.35; 99% CI 4.19–4.52) and dementia (aOR 2.02; 99% CI 1.90–2.15) were associated with the highest aOR (Table 2). Compared to patients without hospital readmission, one-year mortality was higher for patients having sepsis-related hospital readmission (aOR 2.93; 99% CI 2.78–3.09) than for those with hospital readmission for other acute care (aOR 1.25; 99% CI 1.20–1.30). ICU admission was inversely associated with 1-year mortality, whether septic shock was present or not. Compared to patients without ICU admission and no septic shock, the aOR of patients without ICU admission and septic shock was higher (aOR 1.82; 99% CI 1.63–2.04) (see Additional file 1: eTable 6 for patients characteristics). Compared to urinary and genital tracts infection, skin and soft tissues, bacteremia, heart and mediastinum, multiple sites of infection and lower respiratory tract infection had the highest aOR. The directionality of the risk factors was similar when the analysis was restricted to explicit sepsis (see Additional file 1: eTable 7) or with the Cox regression (see Additional file 1: eTable 8). Additionally, 1-year Kaplan–Meier crude survival curve illustrates the difference in survival between patients with and without 1-year hospital readmission for recurrent sepsis (see Additional file 1: eFig. 1).

## Discussion

### Hospital readmission and associated mortality

Our study confirmed that hospital readmission is highly expected for most of patients hospitalized with sepsis. More than 70% of the patients who survived a first episode of sepsis experienced hospital readmission in acute care in the following year, of whom 18.1% had readmission for recurrent sepsis. One-year hospital readmission has been rarely assessed in previous studies. In previous works, 29.7 and to 42.7% of sepsis patients experienced all causes 90-days readmission and 22 to 57.4% experienced all causes readmission at 1 year [4, 7, 13, 33–35]. Indeed, numerous patients have comorbidities and a new admission in MSO is expected. We report a slightly higher readmission rate, but we have considered all hospital readmissions without exclusion criteria. A few studies report a high proportion (38.0 to 61.2%) of hospital readmission specifically due to infection (including sepsis) [13, 35], but there is a lack of data in the literature regarding 1-year readmission specifically due to sepsis. In line with previous studies, the mortality rate was twice higher for patients who experienced hospital readmission for recurrent sepsis compared to the patients without hospital readmission or re-hospitalized for other causes (39.8% vs 19.6%) [6, 36].

### Risk factors for recurrent sepsis

Risk factors for patients with recurrent sepsis, compared to patients not re-hospitalized or re-hospitalized for other causes, were identified. In line with previous studies [1, 6, 8, 10, 10, 11, 33, 37–41], older patients and patients with comorbidities, especially renal disease and cancer, had the greatest odds of hospital readmission for recurrent sepsis. Indeed, immunosenescence of old patients but also cancer and associated medical procedures which impair the immune system, may increase the risk of relapse or new sepsis episode [8]. In addition, patients who had longer hospitalization or sepsis due to heart and mediastinum infection were at higher risk of hospital readmission and rehospitalization in MSO in the following 12 months for acute cardiac events were also more frequent for patients with recurrent sepsis. Indeed, longer hospitalization can act as proxy for the most serious sepsis cases or debilitated patients and infective endocarditis are frequent pathology in the elderly and associated with unfavorable outcomes [1, 6, 12, 42–46]. Moreover, the association between sepsis survivors and cardiovascular problem have been previously identified [47]. Hospital discharge to long-term care appeared as a protective factor in our study, possibly reflecting a limitation to acute care readmission for older and debilitated patients preferably referred to hospice care, or a larger use of follow-up care and rehabilitation contributing to better patient recovery [1, 6]. Discharge to acute care, appears to be a protective factor for readmission for recurrent sepsis compared to readmission for other causes, probably because such patients are referred post-sepsis to a unit for management of a pre-existing a primary health condition, and such patients tend to be re-hospitalized for this primary condition instead of sepsis.

### Recurrent sepsis and other risk factors for 1-year mortality

Recurrent sepsis emerged as an important risk factor for 1-year mortality (~ threefold increase in 1-year mortality), along with five others important risk factors: age, cancer, dementia, septic shock and skin and soft tissues infection (> 1.5-fold increase in 1-year mortality). Indeed, readmission for sepsis was associated with higher risk of 30-days, 6-month and 2-years mortality in previous studies [6, 36]. Apart from recurrent sepsis, age, dementia and cancer, which usually result in complicated clinical scenario, represented important risk factors [23, 48–50]. Cancer is also an important risk factor in middle-aged patients [14, 37, 51, 52]. However, the immunosenescence in older patients might be comparable to the immune impairment of patients with cancer or receiving cancer treatment [37]. Dementia is also particularly present in older patients hospitalized with sepsis and tends to adversely influence clinical outcomes [49]. Finally, the higher odds of mortality identified for skin and soft tissues infection may be related to the most common etiologies of such infections, possibly reflecting the high frequency of *Staphylococcus aureus* infection, often associated with shock and mortality [45, 53]. The protective effect of ICU admission, with an increased case fatality rate for septic patients not admitted to ICU, was also noted in a previous study in France. ICU access is part of the strategy to tackle sepsis in the “surviving sepsis campaign” [54]. While admission to ICU for sepsis is usual but not mandatory, lack of admission for septic shock can be explained by several hypothesis as misdiagnosis, suboptimal care and, more probably, limitations to ICU referral of older patients [18, 54].

### Preventable and non-preventable risk factors

Risk factor analysis are of interest for practitioners or decision makers when preventable risk factors can be identified, allowing subsequent intervention [9]. In previous studies, a low percentage of sepsis-related deaths were estimated preventable, at least in high income countries [55]. Moreover, medico-administrative databases, as the one used in this study, can lack of detailed information regarding these potentially preventable risks. However, some factors suggest a room for consideration and possible action. Although non-modifiable, some risk factors can act as indicators, raising awareness on specific patients in order to adapt their care pathway during their hospital stay or after their hospital discharge [9]. Because recurrent sepsis was one of main risk factor for mortality, preventing and reducing hospital readmission for recurrent sepsis could participate at reducing the mortality rate. Specific acute and post-acute care interventions could be implemented for patients at risk of recurrent sepsis, including patients with comorbidities (especially cancer), the elderly, patients with cardiac infection or more generally sepsis that have required long hospital stay. Indeed, a specific clinical pathway for sepsis according to patients profile was suggested in order to improve patients outcomes and has been already implemented for patients with cancer in Australia [3, 56]. Regarding post-acute care, discharge to long-term care appeared to be a protective factor for recurrent sepsis in our study. This suggests, as previously mentioned, fewer readmission to acute care for the most debilitated patients transferred to palliative care or, in line with previous studies, a real protective effect of post-sepsis rehabilitation [1, 6, 8, 57]. The protective effect of long-term care could suggests possible need for more transfer to long-term care or closer follow-up of some patients returning home and should be explored in additional research [8, 9]. Regarding acute care, the use of ICU appeared to be a protective factor for mortality and recurrent sepsis (in the absence of septic shock) as suggested by a previous study in France [18]. However, the higher risk of recurrent sepsis for patients with cardiac and mediastinum infection or the higher risk of mortality for skin and soft tissue infection should call for special attention or intervention, even for patients not admitted to ICU. Indeed, comorbidities might transform mild skin infection in life-threatening infection and antimicrobial resistance is relatively common in this infection sites [58, 59]. Infective endocarditis is common in the elderly due to the increasing number of cardiac procedures and the recommended therapeutic strategy can also be complicated in these patients, putting them at risk of recurrent sepsis or death [46]. Despite possible improvements, some important risk factors for recurrent sepsis or mortality can be considered as non or moderately preventable, as age or comorbidities [3, 55]. Indeed, the severity of associated illnesses at sepsis onset could make unlikely patient survival even with effective sepsis treatment [55]. However, further studies regarding the care pathway of sepsis patients and more personalized sepsis management and post-acute care according to the different typologies of patients based on age, comorbidities, sites of infection or severity of the sepsis onset could potentially improve patient outcomes, survival and cost for the health care system [8, 12, 56, 60]. General international guidelines are regularly established for sepsis management [61]. In France, the French National Authority for Health (HAS) is currently building integrated recommendations for sepsis management [62]. Based on the results of this study, specific recommendations could be beneficial for patients at high risk of recurrent sepsis.

### Limitations of the study

This study is based on a secondary data analysis of medico-administrative data, without possible validation of sepsis cases based on clinical data. As a result, patients hospitalized with sepsis, based on a combination of implicit and explicit sepsis, could be slightly over or underestimated [30, 63, 64]. However, the analyses, based on explicit sepsis only, confirmed the robustness of our results. Due to administrative and regulation hurdles, a database with only sepsis with bacterial etiology was used in this analysis. However, fungus or viruses represents a small percentage of all sepsis causative pathogens [45]. Moreover, regarding recurrent sepsis, the difference between new pathogens or relapse of the initial sepsis with the same pathogens could not be made as the pathogens, potential antibiotic resistance and the connection between pathogen and infection site could not be identified. Finally, the absence of information of physical and mental impairments after discharge should be further considered in a study of the care pathway of these patients.

## Conclusion

Hospital readmission and mid-term mortality of patients hospitalized with sepsis are substantial. In our study, patients with cancer and recurrent sepsis had the highest odds of 1-year mortality. Our study suggests that some risk factors, even if non modifiable, should be considered to personalize the care pathway of the most vulnerable patients. Moreover, the need for post-acute care and adequate follow-up after hospital discharge should be further considered. Changes in the care pathway to reduce sepsis related readmission could improve survival and associated economic burden for the health care system.

## Supplementary Information

**Additional file 1. eMethods** Description of the French National Hospital Discharge Database (PMSI) and Inter-Scheme consumption data (DCIR), **eTable 1** ICD-10 codes used to identify sepsis of presumed bacterial etiology according to type of selection in sepsis patients  >  15 years, **eTable 2** Description of the variables, **eTable 3** Risk factors, recorded during the index hospitalization, for 1-year hospital readmission for recurrent sepsis for 140,658 adult patients hospitalized with explicit sepsis in metropolitan France in 2018: univariate and multivariate logistic regressions, **eTable 4** Risk factors, recorded during the index hospitalization, for 1-year hospital readmission for recurrent sepsis for 131,364 adult septic patients who were rehospitalized (with sepsis or other causes) in the following year, metropolitan France, 2018: univariate and multivariate logistic regressions, **eTable 5** Comorbidities and demographic, hospital stay and infection characteristics of adult patients with sepsis according to 1-year survival, metropolitan France 2018, **eTable 6** Charlson index and demographic of adult patients with septic choc without ICU admission, metropolitan France 2018, **eTable 7** Risk factors for 1-year mortality of the 140,658 adult patients surviving a first episode of sepsis (explicit sepsis only) in metropolitan France in 2018: univariate and multivariate logistic regression, **eTable 8** Cox regression and hazard ratio for 1-year mortality of the 178,017 adult patients surviving a first episode of sepsis in metropolitan France in 2018: Multivariate analysis.

## Data Availability

The data that support the findings of this study are available from the French national health insurance information system but restrictions apply to the availability of these data, which were used under license for the current study, and so are not publicly available.
