# Supplementary material for: One-year hospital readmission for recurrent sepsis: associated risk factors and impact on 1-year mortality—a French nationwide study
Source: Crit Care. 2022 Nov 29;26:371. doi: 10.1186/s13054-022-04212-9 (PMC9710072; doi:10.1186/s13054-022-04212-9)
Supplement: Supplementary file 1 — Additional file 1. eMethods Description of the French National Hospital Discharge Database (PMSI) and Inter-Scheme consumption data (DCIR), eTable 1 ICD-10 codes used to identify sepsis of presumed bacterial etiology according to type of selection in sepsis patients > 15 years, eTable 2 Description of the variables, eTable 3 Risk factors, recorded during the index hospitalization, for 1-year hospital readmission for recurrent sepsis for 140,658 adult patients hospitalized with explicit sepsis in metropolitan France in 2018: univariate and multivariate logistic regressions, eTable 4 Risk factors, recorded during the index hospitalization, for 1-year hospital readmission for recurrent sepsis for 131,364 adult septic patients who were rehospitalized (with sepsis or other causes) in the following year, metropolitan France, 2018: univariate and multivariate logistic regressions, eTable 5 Comorbidities and demographic, hospital stay and infection characteristics of adult patients with sepsis according to 1-year survival, metropolitan France 2018, eTable 6 Charlson index and demographic of adult patients with septic choc without ICU admission, metropolitan France 2018, eTable 7 Risk factors for 1-year mortality of the 140,658 adult patients surviving a first episode of sepsis (explicit sepsis only) in metropolitan France in 2018: univariate and multivariate logistic regression, eTable 8 Cox regression and hazard ratio for 1-year mortality of the 178,017 adult patients surviving a first episode of sepsis in metropolitan France in 2018: Multivariate analysis. [file 13054_2022_4212_MOESM1_ESM.docx]

**eMethods: Description of the French National Hospital Discharge Database (PMSI) and Inter-Scheme consumption data (DCIR).**

The study, analysis and data extraction were approved by the French Data Protection Agency (CNIL, approval DE-2016–176). Informed consent is waived for the use of these anonymised secondary data, as mentioned in the Social Security Code, Article L161–28-1. All methods were performed in accordance with CNIL regulations and with REporting of studies Conducted using Observational Routinely collected Data (RECORD) guideline**.** The **National Health Data System** (Système National des Données de Santé: SNDS) essentially contains individual data used for billing and reimbursement of outpatient health care consumption (Inter-Scheme consumption data: DCIR) and private and public hospital data (Medical Information System Programme: PMSI) collected by the Agence technique de l’information sur l’hospitalisation (ATIH)^1^. Regarding sepsis selection in the PMSI, sepsis was identified as the combination of explicit and implicit sepsis. The selection of septic patients in the medico-administrative database is based on a translation of the Sepsis-3 definition^1^ . Explicit sepsis was defined as a stay for which sepsis explicitly appeared in the ICD-10 codes (e.g., A40: streptococcal sepsis) as primary diagnosis (PD: condition requiring hospitalization), related diagnosis (RD: adds information to PD) or significant associated diagnosis (SAD: complications and co-morbidities potentially affecting the course or cost of hospitalization). In the absence of specific sepsis ICD-10 codes, implicit sepsis was defined as a stay with one of the selected ICD-10 codes for infection as PD, RD or SAD, with two associated conditions: ICU admission and one of the selected ICD-10 codes for organ dysfunction or a code for organ support from the Common Classification of Medical Acts (CCAM). The lists of ICD-10 codes and codes for organ dysfunction or support were established according to previous publications and based on medical and epidemiological expertise. Before the study population selection, sepsis-related hospital stays with an interval between discharge and admission <1days or an interval between discharge and admission between 1 and 7 days with the same primary diagnosis or bacteremia as primary diagnosis for the subsequent hospital stay were considered as the same hospital stay and grouped together.

**eTable 1**. ICD-10 codes used to identify sepsis of presumed bacterial etiology according to type of selection in sepsis patients >15yrs.

| Explicit sepsis codes*^a,b,d^* | Implicit sepsis *^b,c,d^* | | |
| --- | --- | --- | --- |
|  | **Infection codes*^a^*** | **1^st^ associated condition** | **2^nd^ associated condition** |
| Sepsis of presumed bacterial etiology | |  |  |
| A02.1, A40.0-A40.9, A41.0-A41.9, A48.0, A48.3, O85, O88.3, P36.00, P36.10, P36.20, P36.30, P36.40, P36.50, P36.80, P36.90, R57.2, R57.8, R65.1 | A04.0-A04.9, A39.0-A39.9, G00.0-G00.9, I33.0, J06.8, J13, J14, J15.0-J15.9, J16.0-J16.8, J18.0-J18.9, J86.9, K65.0, K65.9, K81.0, K83.0, L02.2, L08.9, M00.0-M00.99, M46.20-M46.29, M60.00-M60.09, M86.00-M86.09, M86.90-M86.99, N13.6, N39.0, P00.2, T79.3, T80.2, T81.1, T81.4, T82.7, T84.5, T85.7 | ICU admission | ICD-10 codes for organ dysfunction : A483, D65, D689, D695, D696, D762, E86, E872, F05.0-F05.9, F09, G934, I460, I469, I950, I959, J80, J81, J952, J969, K72.0, K72.9, N08.0, N08.8, N16.0, N17.0-N17.9, N19, R09.2, R17.0-17.9, R34, R40.0-R40.28, R39.2, R41.0, R41.8, R55, , R57.1, R57.9  CCAM codes for organ support: DKMD001, DKMD002, EQLF002, EQLF003, FELF003, GLLD003, GLLD004, GLLD008, GLLD012, GLLD015, GLLD019, JVJB002, JVJF002, JVJF003, JVJF005, |

^a^ One of the ICD-10 code as primary diagnosis (PD: condition requiring hospitalization), related diagnosis (RD: adds information to PD) or significant associated diagnosis (SAD: complications and co-morbidities potentially affecting the course or cost of hospitalization)

.^b^ Sepsis = sepsis explicit sepsis + implicit sepsis

^c^ Implicit sepsis= ICD-10 code of infection + ICU admission+ organ dysfunction/support

^d^ Stays shorter than 24h hours without death were excluded from our selection

**eTable 2.** Description of the variables.

| **Variables** | **Sub-categories** |
| --- | --- |
| 30-day mortality | Yes (In-hospital mortality and mortality outside of the hospital during the 30 days following the discharge from the index hospitalization with sepsis), No (Patient still alive at 30 days following the discharge from the index hospitalization with sepsis) |
| 90-day mortality | Yes (In-hospital mortality and mortality outside of the hospital during the 90 days following the discharge from the index hospitalization with sepsis), No (Patient still alive at 90 days following the discharge from the index hospitalization with sepsis) |
| 6-month mortality | Yes (In-hospital mortality and mortality outside of the hospital during the 6 months following the discharge from the index hospitalization with sepsis), No (Patient still alive 6 months following the discharge from the index hospitalization with sepsis) |
| 1-year mortality | Yes (In-hospital mortality and mortality outside of the hospital during the year following the discharge from the index hospitalization with sepsis), No (Patient still alive 12 months following the discharge from the index hospitalization with sepsis) |
| Hospital readmission | Acute care^c^ (at least one readmission in acute care ward for other causes than sepsis in the year following the discharge from the index hospitalization with sepsis), Sepsis recurrence (Sepsis-related hospital readmission in acute-care ward in the year following the discharge from the index hospitalization, with or without other hospital readmission in acute care ward for other causes), No (No readmission in acute care in the year following the discharge from the index hospitalization with sepsis) |
| Gender | Male, Female |
| Age | 16-25, 26-35, 36-45, 46-55, 56-65, 66-75, 75-85, >85 |
| Charlson Index | 0, 1-2, 3-4, ≥5 based on the modified classification of Quan et al. (2011)^2,^*^c^* |
| Comorbidities | Heart failure, Dementia, Chronic pulmonary diseases, Liver disease (mild, moderate or severe liver diseases), Diabetes with chronic complications, Paraplegia et hemiplegia, Renal disease, Cancer (cancer or metastatic carcinoma), AIDS/HIV. Based on the modified classification of Quan and al. (2011)^2,^*^b^* |
| Admission source | Acute care (From a short hospital stay in medicine, surgery or obstetrics ward, after a transfer for or after a medical procedure or from psychiatry unit), Long term care (From follow-up and rehabilitation care unit or from long term care unit or home care), Home |
| Hospital discharge | Acute care (To a short hospital stay in medicine, surgery or obstetrics units, after a transfer for or after a medical procedure or from psychiatry unit), Long term care (To follow-up and rehabilitation care unit or from long term care unit or home care), Home, Death. |
| Length of stay (days) | Number of days from date of admission to date of discharge, further stratified in 4 groups <7days, 7-14 days, 15-30 days, >30 days |
| ICU admission*^c^* and septic shock*^d^* | No ICU admission and septic shock, No UCI admission and no septic shock, ICU admission and septic shock or ICU admission and no septic shock during the index hospitalization |
| Infection site | Lower respiratory tract, Urinary and genital tracts, Abdomen and digestive tract, Heart and mediastinum, Skin and soft tissues, Associated with medical device, Bones and joints, Nervous system, Ears nose and throat, Infections during pregnancy, Eyes, Multiple site, unknown (Sepsis without primary site identified: primary bacteremia or sepsis with no infection site recorded)^3^. |

^a^ *In case of hospital readmission for sepsis and other diagnosis, sepsis-related hospitalization was prioritized*

*^b^ classification cancer et metastatic carcinoma were classified as cancer, mild, severe liver disease were classified as liver disease.*

^c^ ICU admission: recorded in one of the following medical unit: Intensive care unit (ICU), Pediatric ICU, Neonatal ICU, Other ICU, Coronary care unit, Neuro-intensive care; No ICU admission: not recorded in one of the above listed units

*^d^ Septic shock: ICD-10 codes R57.2, R57.8 as primary diagnosis, related diagnosis or significant associated diagnosis, No septic shock: No ICD-10 codes R57.2, R57.8 as primary diagnosis, related diagnosis or significant associated diagnosis*

**eTable 3.** - Risk factors, recorded during the index hospitalization, for 1-year hospital readmission for recurrent sepsis for 140658 adult patients hospitalized with explicit sepsis in metropolitan France in 2018: univariate and multivariate logistic regressions.

|  | **cOR^1^ (99% CI)** | | | **Pvalue** | **aOR^1^ (99% CI)** | | | **Pvalue** |
| --- | --- | --- | --- | --- | --- | --- | --- | --- |
| **Patient characteristics** | | | | | | | |  |
| **Sex (ref=men)** | 0.73 | (0.70 | - 0.76) | <0.001 | 0.84 | (0.80 | - 0.87) | <0.001 |
|  |  |  |  |  |  |  |  |  |
| **Age (ref=16-30)** |  |  |  | <0.001 |  |  |  | <0.001 |
| 31-45 | 1.39 | (1.19 | - 1.63) |  | 1.19 | (1.01 | - 1.39) |  |
| 46-55 | 1.84 | (1.59 | - 2.13) |  | 1.30 | (1.12 | - 1.51) |  |
| 56-65 | 2.20 | (1.92 | - 2.53) |  | 1.46 | (1.27 | - 1.68) |  |
| 66-75 | 2.28 | (1.99 | - 2.61) |  | 1.52 | (1.32 | - 1.74) |  |
| 76-85 | 1.81 | (1.58 | - 2.07) |  | 1.33 | (1.16 | - 1.54) |  |
| >85 | 1.26 | (1.10 | - 1.45) |  | 1.07 | (0.92 | - 1.24) |  |
|  |  |  |  |  |  |  |  |  |
| **Heart failure (ref=no)** | 1.10 | (1.04 | - 1.15) | <0.001 | 1.13 | (1.07 | - 1.20) | <0.001 |
| **Dementia (ref=no)** | 0.64 | (0.58 | - 0.70) | <0.001 | 0.82 | (0.74 | - 0.90) | <0.001 |
| **Chronic pulmonary disease^2^ (ref=no)** | 1.06 | (1.00 | - 1.13) | 0.02 | ─ |  |  |  |
| **Rheumatologic disease (ref=no)** | 1.21 | (1.04 | - 1.41) | 0.03 | 1.29 | (1.09 | - 1.53) | <0.001 |
| **Liver disease (ref=no)** | 1.44 | (1.33 | - 1.56) | <0.001 | 1.33 | (1.22 | - 1.44) | <0.001 |
| **Diabetes with chronic complications (ref=no)** | 1.44 | (1.34 | - 1.56) | <0.001 | 1.28 | (1.18 | - 1.39) | <0.001 |
| **Paraplegia and hemiplegia^2^ (ref=no)** | 1.05 | (0.96 | - 1.15) | 0.16 | ─ |  |  |  |
| **Renal disease (ref=no)** | 1.46 | (1.39 | - 1.55) | <0.001 | 1.50 | (1.42 | - 1.59) | <0.001 |
| **Cancer (ref=no)** | 2.16 | (2.07 | - 2.25) | <0.001 | 1.98 | (1.89 | - 2.07) | <0.001 |
| **AIDS HIV (ref=no)** | 1.40 | (1.07 | - 1.81) | 0.001 | 1.35 | (1.03 | - 1.78) | 0.005 |
| **Hospital stay characteristics** | | | | | | | |  |
| **Hospital discharge(ref=Home)** |  |  |  | <0.001 |  |  |  | <0.001 |
| Acute care | 1.10 | (1.03 | - 1.16) |  | 1.07 | (1.00 | - 1.13) |  |
| Home care | 1.45 | (1.25 | - 1.69) |  | 1.06 | (0.91 | - 1.23) |  |
| Long-term care | 0.95 | (0.90 | - 1.00) |  | 0.88 | (0.83 | - 0.93) |  |
| **Length of stay (in days) (ref≤7)** |  |  |  | <0.001 |  |  |  | <0.001 |
| 7-30 | 1.34 | (1.26 | - 1.43) |  | 1.19 | (1.35 | - 1.59) |  |
| 31-90 | 1.84 | (1.71 | - 1.98) |  | 1.47 | (1.12 | - 1.27) |  |
| >90 | 2.23 | (1.92 | - 2.58) |  | 1.90 | (1.62 | - 2.23) |  |
| **ICU admission and septic shock (ref=absence of both)** |  |  |  | <0.001 |  |  |  | 0.011 |
| No ICU admission and septic shock | 0.82 | (0.71 | - 0.96) |  | 0.91 | (0.78 | - 1.07) |  |
| ICU admission and septic shock | 1.07 | (1.01 | - 1.12) |  | 0.97 | (0.91 | - 1.03) |  |
| ICU admission and no septic shock | 1.10 | (1.05 | - 1.16) |  | 0.94 | (0.89 | - 0.99) |  |
| **Infection characteristics** | | | | | | | |  |
| **Site (ref=Urinary and genital tracts)** |  |  |  | <0.001 |  |  |  | <0.001 |
| Gastrointestinal and abdomen | 1.27 | (1.16 | - 1.39) |  | 1.14 | (1.04 | - 1.25) |  |
| Primary bacteraemia | 1.45 | (1.36 | - 1.55) |  | 1.24 | (1.16 | - 1.32) |  |
| Bones and joints | 1.09 | (0.94 | - 1.25) |  | 1.01 | (0.88 | - 1.17) |  |
| Heart and mediastinum | 2.01 | (1.84 | - 2.20) |  | 1.48 | (1.35 | - 1.62) |  |
| Multiple | 1.44 | (1.35 | - 1.54) |  | 1.14 | (1.06 | - 1.22) |  |
| Material device | 1.73 | (1.55 | - 1.93) |  | 1.21 | (1.08 | - 1.36) |  |
| Lower respiratory tract | 1.04 | (0.97 | - 1.12) |  | 0.95 | (0.88 | - 1.03) |  |
| Skin and soft tissues | 1.35 | (1.23 | - 1.58) |  | 1.21 | (1.10 | - 1.34) |  |
| Others | 0.44 | (0.37 | - 0.53) |  | 0.52 | (0.43 | - 0.62) |  |

*^1^cOR: Crude odds ratio; aOR: Adjusted odds ratio*; ^2^Variables excluded from multivariate analysis.

**eTable 4.** Risk factors, recorded during the index hospitalization, for 1-year hospital readmission for recurrent sepsis for 131364 adult septic patients who were rehospitalized (with sepsis or other causes) in the following year, metropolitan France, 2018: univariate and multivariate logistic regressions.

|  | **cOR^1^ (99% CI)** | | | **Pvalue** | **aOR^1^ (99% CI)** | | | **Pvalue** |
| --- | --- | --- | --- | --- | --- | --- | --- | --- |
| **Patient characteristics** | | | | | | | |  |
| **Sex (ref=men)** | 0.83 | (0.79 | - 0.86) | <0.001 | 0.88 | (0.85 | - 0.92) | <0.0001 |
|  |  |  |  |  |  |  |  |  |
| **Age (ref=16-30)** |  |  |  | <0.001 |  |  |  | <0.001 |
| 31-45 | 1.21 | (1.05 | - 1.40) |  | 1.13 | (0.98 | - 1.31) |  |
| 46-55 | 1.38 | (1.20 | - 1.58) |  | 1.18 | (1.03 | - 1.36) |  |
| 56-65 | 1.60 | (1.41 | - 1.82) |  | 1.32 | (1.16 | - 1.50) |  |
| 66-75 | 1.65 | (1.46 | - 1.88) |  | 1.37 | (1.21 | - 1.56) |  |
| 76-85 | 1.42 | (1.25 | - 1.62) |  | 1.26 | (1.10 | - 1.43) |  |
| >85 | 1.21 | (1.06 | - 1.38) |  | 1.15 | (1.00 | - 1.32) |  |
|  |  |  |  |  |  |  |  |  |
| **Heart failure^2^ (ref=no)** | 1.04 | (0.99 | - 1.08) | 0.042 | ─ |  |  |  |
| **Dementia (ref=no)** | 0.86 | (0.78 | - 0.95) | <0.001 | 0.96 | (0.87 | - 1.06) | 0.325 |
| **Chronic pulmonary disease^2^ (ref=no)** | 1.02 | (0.96 | - 1.08) | 0.467 | ─ |  |  |  |
| **Rheumatologic disease^2^ (ref=no)** | 1.14 | (0.98 | - 1.33) | 0.026 | ─ |  |  |  |
| **Liver disease (ref=no)** | 1.31 | (1.21 | - 1.41) | <0.001 | 1.25 | (1.16 | - 1.35) | <0.001 |
| **Diabetes with chronic complications (ref=no)** | 1.32 | (1.23 | - 1.42) | <0.001 | 1.39 | (1.32 | - 1.47) | <0.001 |
| **Paraplegia and hemiplegia^2^ (ref=no)** | 0.98 | (0.90 | - 1.06) | 0.43 | ─ |  |  |  |
| **Renal disease (ref=no)** | 1.36 | (1.29 | - 1.43) | <0.001 | 1.39 | (1.32 | - 1.47) | <0.001 |
| **Cancer (ref=no)** | 1.69 | (1.62 | - 1.75) | <0.001 | 1.59 | (1.52 | - 1.66) | <0.001 |
| **AIDS HIV (ref=no)** | 1.31 | (1.02 | - 1.69) | 0.001 | 1.29 | (1.00 | - 1.67) | 0.011 |
| **Hospital stay characteristics** | | | | | | | |  |
| **Hospital discharge(ref=Home)** |  |  |  | <0.001 |  |  |  | <0.001 |
| Acute care | 0.77 | (0.73 | - 0.81) |  | 0.83 | (0.79 | - 0.88) |  |
| Home care | 1.39 | (1.20 | - 1.61) |  | 1.11 | (0.96 | - 1.29) |  |
| Long-term care | 0.90 | (0.86 | - 0.95) |  | 0.88 | (0.84 | - 0.93) |  |
| **Length of stay (in days) (ref≤7)** |  |  |  | <0.001 |  |  |  | <0.001 |
| 7-30 | 1.24 | (1.17 | - 1.31) |  | 1.16 | (1.09 | - 1.23) |  |
| 31-90 | 1.55 | (1.45 | - 1.66) |  | 1.45 | (1.34 | - 1.56) |  |
| >90 | 1.89 | (1.64 | - 2.17) |  | 1.91 | (1.65 | - 2.22) |  |
| **ICU admission and septic shock (ref=absence of both)** | |  |  | <0.001 |  |  |  | 0.011 |
| No ICU admission and septic shock | 0.87 | (0.74 | - 1.02) |  | 0.96 | (0.82 | - 1.13) |  |
| ICU admission and septic shock | 0.96 | (0.91 | - 1.01) |  | 0.96 | (0.90 | - 1.02) |  |
| ICU admission and no septic shock | 0.82 | (0.78 | - 0.85) |  | 0.83 | (0.79 | - 0.87) |  |
| **Infection characteristics** | | | | | | | |  |
| **Site (ref=Urinary and genital tracts)** |  |  |  | <0.001 |  |  |  | <0.001 |
| Gastrointestinal and abdomen | 1.16 | (1.06 | - 1.26) |  | 1.11 | (1.01 | - 1.21) |  |
| Primary bacteraemia | 1.37 | (1.29 | - 1.46) |  | 1.24 | (1.16 | - 1.32) |  |
| Bones and joints | 1.09 | (0.95 | - 1.25) |  | 1.07 | (0.93 | - 1.23) |  |
| Heart and mediastinum | 1.57 | (1.44 | - 1.72) |  | 1.34 | (1.22 | - 1.46) |  |
| Multiple | 1.24 | (1.17 | - 1.32) |  | 1.10 | (1.03 | - 1.18) |  |
| Material device | 1.21 | (1.09 | - 1.34) |  | 1.03 | (0.93 | - 1.14) |  |
| Lower respiratory tract | 0.93 | (0.87 | - 0.99) |  | 0.97 | (0.91 | - 1.04) |  |
| Skin and soft tissues | 1.39 | (1.27 | - 1.53) |  | 1.29 | (1.17 | - 1.42) |  |
| Others | 0.61 | (0.51 | - 0.72) |  | 0.66 | (0.56 | - 0.79) |  |

*^1^cOR: Crude odds ratio; aOR: Adjusted odds ratio*; ^2^Variables excluded from multivariate analysis.

**eTable 5.** Comorbidities and demographic, hospital stay and infection characteristics of adult patients with sepsis according to 1-year survival, metropolitan France 2018.

|  | **1-year survival** | | **Death in the following year** | |
| --- | --- | --- | --- | --- |
|  | **(N=138321)** | | **(N=39696)** | |
|  | **N** | **%** | **N** | **%** |
| **Patient characteristics***^a^* | | | |  |
| **Sex** |  |  |  |  |
| Men | 77831 | 56.3 | 23083 | 58.2 |
| Women | 60490 | 43.7 | 16613 | 41.9 |
| **Age *median (IQR)*** | *69(57-80)* |  | *77(66-85)* |  |
| 16-30 | 6532 | 4.7 | 260 | 0.7 |
| 31-45 | 10599 | 7.7 | 930 | 2.3 |
| 46-55 | 14087 | 10.2 | 2315 | 5.8 |
| 56-65 | 25153 | 18.2 | 5769 | 14.5 |
| 66-75 | 33743 | 24.4 | 9569 | 24.1 |
| 76-85 | 30634 | 22.2 | 11049 | 27.8 |
| >85 | 17573 | 12.7 | 9804 | 24.7 |
| **Charlson**^1^**. me*dian (IQR)*** | *1(0-2)* |  | *2(2-6)* |  |
| 0 | 58954 | 42.6 | 7018 | 17.7 |
| 1-2 | 50437 | 36.5 | 14174 | 35.7 |
| 3-4 | 17735 | 12.8 | 7281 | 18.3 |
| >=5 | 11195 | 8.1 | 11223 | 28.3 |
| **Comorbidities**^1^ |  |  |  |  |
| Heart failure | 26763 | 19.4 | 9888 | 24.9 |
| Dementia | 5620 | 4.1 | 3684 | 9.3 |
| Chronic pulmonary disease | 15442 | 11.2 | 4850 | 12.2 |
| Rheumatologic disease | 1749 | 1.3 | 499 | 1.3 |
| Liver disease | 6907 | 5.0 | 2254 | 5.7 |
| Diabetes with chronic complications | 8148 | 5.9 | 2499 | 6.3 |
| Paraplegia and hemiplegia | 7409 | 5.4 | 2247 | 5.7 |
| Renal disease | 16163 | 11.7 | 6407 | 16.1 |
| Cancer | 23204 | 16.8 | 16982 | 42.8 |
| AIDS HIV | 661 | 0.5 | 110 | 0.3 |
| **Hospital stay characteristics***^a^* | | | | |
| **Admission source** |  |  |  |  |
| Acute care | 11634 | 8.4 | 3859 | 9.7 |
| Home | 124821 | 90.2 | 34716 | 87.5 |
| Home care | 132 | 0.1 | 194 | 0.5 |
| Long term care | 1734 | 1.3 | 927 | 2.3 |
| **Hospital discharge** |  |  |  |  |
| Acute care | 20169 | 14.6 | 7429 | 18.7 |
| Home | 91830 | 66.4 | 22263 | 56.1 |
| Home care | 1195 | 0.9 | 1025 | 2.6 |
| Long term care | 25127 | 18.2 | 8979 | 22.6 |
| **Length of stay. days** |  |  |  |  |
| <7 | 20359 | 14.7 | 4314 | 10.9 |
| 7-30 | 23180 | 16.8 | 8302 | 20.9 |
| 31-90 | 92772 | 67.1 | 26440 | 66.6 |
| >90 | 2010 | 1.5 | 640 | 1.6 |
| **ICU admission and septic shock** |  |  |  |  |
| No ICU admission and septic shock | 1650 | 1.2 | 1117 | 2.8 |
| No ICU admission and no septic shock | 59809 | 43.2 | 21217 | 53.5 |
| ICU admission and septic shock | 19303 | 14.0 | 4644 | 11.7 |
| ICU admission and no septic shock | 57559 | 41.6 | 12718 | 32.0 |
| **Site of infection***^a^* |  |  |  |  |
| Gastrointestinal and abdomen | 9281 | 6.7 | 2046 | 5.2 |
| Primary bacteraemia | 21442 | 15.5 | 7795 | 19.6 |
| Bones and joints | 3356 | 2.4 | 526 | 1.3 |
| Heart and mediastinum^1^ | 5936 | 4.3 | 2128 | 5.4 |
| Multiple | 29723 | 21.5 | 9474 | 23.9 |
| Medical device | 5099 | 3.7 | 1558 | 3.9 |
| Lower respiratory tract | 27287 | 19.7 | 7100 | 17.9 |
| Skin and soft tissues | 5914 | 4.3 | 2386 | 6.0 |
| Urinary and genital tracts | 25640 | 18.5 | 6210 | 15.6 |
| Others | 4643 | 3.4 | 473 | 1.2 |
| **1-year hospital readmission***^b^* |  |  |  |  |
| Acute care (excluding sepsis) | 84847 | 61.3 | 22789 | 57.4 |
| Recurrent sepsis | 14281 | 10.3 | 9447 | 23.8 |
| None | 39193 | 28.3 | 7460 | 18.8 |

*^a^During index hospitalization*

*^b^During the one-year period following the index sepsis-related hospital discharge*

**eTable 6.** Demographic characteristics and Charlson index of adult patients with septic choc and no ICU admission, metropolitan France 2018.

| **Variables** | **N** | **%** |  |
| --- | --- | --- | --- |
|  |  |  |  |
|  |  |  |  |
| **Sex** |  |  |  |
| Men | 1504 | 54.4 |  |
| Women | 1263 | 45.7 |  |
| **Age, *Median (IQR)*** | *77(69-88)* |  |  |
| 16-30 | 53 | 1.9 |  |
| 31-45 | 83 | 3.0 |  |
| 46-55 | 111 | 4.0 |  |
| 56-65 | 312 | 11.3 |  |
| 66-75 | 515 | 18.6 |  |
| 76-85 | 736 | 26.6 |  |
| >85 | 957 | 34.6 |  |
| **Charlson**^1^**, *Median (IQR)*** | *2(0-3)* |  |  |
| 0 | 1005 | 36.3 |  |
| 1-2 | 981 | 35.5 |  |
| 3-4 | 370 | 13.4 |  |
| >=5 | 411 | 14.9 |  |

**eTable 7.** Risk factors for 1-year mortality of the 140658 adult patients surviving a first episode of sepsis (explicit sepsis only) in metropolitan France in 2018: univariate and multivariate logistic regression.

|  | **cOR1 (99% CI)** | | | **P-value** | **aOR1 (99% CI)** | | | **P-value** |
| --- | --- | --- | --- | --- | --- | --- | --- | --- |
| **Patient characteristics** |  |  |  |  |  |  |  | |
| **Sex (ref=men)** | 0.89 | (0.86 | - 0.92) | <0.001 | 0.96 | (0.92 | - 0.99) | 0.002 |
| **Age (ref=16-30)** |  |  |  | <0.001 |  |  |  | <0.001 |
| 31-45 | 2.34 | (1.90 | - 2.89) |  | 1.99 | (1.60 | - 2.47) |  |
| 46-55 | 4.64 | (3.82 | - 5.64) |  | 3.11 | (2.54 | - 3.80) |  |
| 56-65 | 6.28 | (5.20 | - 7.59) |  | 3.73 | (3.07 | - 4.54) |  |
| 66-75 | 7.65 | (6.35 | - 9.22) |  | 4.64 | (3.82 | - 5.63) |  |
| 76-85 | 9.25 | (7.68 | - 11.15) |  | 6.74 | (5.56 | - 8.19) |  |
| >85 | 13.74 | (11.39 | - 16.56) |  | 11.95 | (9.83 | - 14.53) |  |
| **Heart failure (ref=no)** | 1.43 | (1.37 | - 1.48) | <0.001 | 1.37 | (1.31 | - 1.43) | <0.001 |
| **Dementia (ref=no)** | 2.23 | (2.10 | - 2.36) | <0.001 | 2.00 | (1.87 | - 2.14) | <0.001 |
| **Chronic pulmonary disease (ref=no)** | 1.12 | (1.06 | - 1.18) | <0.001 | 1.16 | (1.09 | - 1.23) | <0.001 |
| **Rheumathologic disease^2^ (ref=no)** | 0.95 | (0.82 | - 1.09) | 0.33 |  |  |  |  |
| **Liver disease (ref=no)** | 1.14 | (1.06 | - 1.23) | <0.001 | 1.57 | (1.45 | - 1.70) | <0.001 |
| **Diabetes with chronic complications^2^ (ref=no)** | 1.03 | (0.96 | - 1.10) | 0.003 |  |  |  |  |
| **Paraplegia and hemiplegia (ref=no)** | 1.11 | (1.04 | - 1.20) | 0.001 | 1.54 | (1.42 | - 1.67) | <0.001 |
| **Renal disease (ref=no)** | 1.38 | (1.32 | - 1.44) | <0.001 | 1.30 | (1.24 | - 1.37) | <.0001 |
| **Cancer (ref=no)** | 3.86 | (3.72 | - 3.99) | <0.001 | 4.73 | (4.54 | - 4.93) | <.0001 |
| **AIDS HIV (ref=no)** | 0.54 | (0.40 | - 0.73) | <0.001 | 0.87 | (0.63 | - 1.19) | 0.25 |
| **Hospital stay characteristics** |  |  |  |  |  |  |  | |
| **Hospital readmission (ref=no hospital readmission)** |  |  |  | <0.001 |  |  |  | <0.001 |
| recurrent sepsis | 3.19 | (3.03 | - 3.36) |  | 2.68 | (2.53 | - 2.84) |  |
| other acute care | 1.37 | (1.32 | - 1.43) |  | 1.20 | (1.15 | - 1.26) |  |
| **ICU admission and septic shock (ref= absence of both)** |  |  |  | <0.001 |  |  |  | <0.001 |
| No ICU admission and septic shock | 1.91 | (1.72 | - 2.11) |  | 1.82 | (1.63 | - 2.04) |  |
| ICU admission and septic shock | 0.68 | (0.65 | - 0.71) |  | 0.81 | (0.77 | - 0.86) |  |
| ICU admission and no septic shock | 0.71 | (0.68 | - 0.74) |  | 0.74 | (0.70 | - 0.77) |  |
| **Infection characteristics** |  |  |  |  |  |  |  | |
| **Site (ref=Urinary and genital tracts)** |  |  |  | <0.001 |  |  |  | <0.001 |
| Gastrointestinal and abdomen | 0.92 | (0.85 | - 0.99) |  | 0.99 | (0.91 | - 1.08) |  |
| Primary bacteremia | 1.46 | (1.39 | - 1.54) |  | 1.41 | (1.33 | - 1.49) |  |
| Bones and joints | 0.60 | (0.52 | - 0.69) |  | 0.84 | (0.72 | - 0.97) |  |
| Heart and mediastinum | 1.57 | (1.46 | - 1.70) |  | 1.42 | (1.30 | - 1.55) |  |
| Multiple | 1.50 | (1.43 | - 1.58) |  | 1.46 | (1.37 | - 1.54) |  |
| Material device | 1.80 | (1.65 | - 1.98) |  | 1.45 | (1.31 | - 1.60) |  |
| Lower respiratory tract | 1.23 | (1.16 | - 1.30) |  | 1.34 | (1.26 | - 1.43) |  |
| Skin and soft tissues | 1.66 | (1.54 | - 1.79) |  | 1.78 | (1.65 | - 1.93) |  |
| Others | 0.40 | (0.35 | - 0.45) |  | 0.95 | (0.82 | - 1.10) |  |

*^1^cOR: Crude odds ratio; aOR: Ajusted odds ratio*; *^2^Variables excluded from multivariate analysis.*

**eTable 8.** Hazard ratio and associated P value for 1-year mortality of the 178017 adult patients surviving a first episode of sepsis in metropolitan France in 2018: Multivariate survival analysis using Cox's regression model.

|  |  | | **Hazard ratio** | | **CI 99%** | **P value** | |
| --- | --- | --- | --- | --- | --- | --- | --- |
| **Patient characteristics** |  |  | |  | |  |  |
| **Sex (ref=men)** |  | 0.99 | | (0,96 - 1,02) | | 0.32 |  |
| **Age (ref=16-30)** | 31-45 | 1.91 | | (1,60 - 2,29) | | <.0001 |  |
|  | 46-55 | 2.86 | | (2,41 - 3,38) | | <.0001 |  |
|  | 56-65 | 3.45 | | (2,93 - 4,06) | | <.0001 |  |
|  | 66-75 | 4.10 | | (3,49 - 4,83) | | <.0001 |  |
|  | 76-85 | 5.50 | | (4,67 - 6,47) | | <.0001 |  |
|  | >85 | 8.39 | | (7,13 - 9,88) | | <.0001 |  |
| **Heart failure (ref=no)** |  | 1.24 | | (1,20 - 1,28) | | <.0001 |  |
| **Dementia (ref=no)** |  | 1.70 | | (1,62 - 1,78) | | <.0001 |  |
| **Chronic pulmonary disease (ref=no)** |  | 1.12 | | (1,07 - 1,16) | | <.0001 |  |
| **Liver disease (ref=no)** |  | 1.36 | | (1,29 - 1,44) | | <.0001 |  |
| **Diabetes with chronic complications (ref=no)** |  | 1.04 | | (0,99 - 1,10) | | 0.046 |  |
| **renal** |  | 1.21 | | (1,17 - 1,26) | | <.0001 |  |
| **Cancer (ref=no)** |  | 3.15 | | (3,06 - 3,24) | | <.0001 |  |
| **AIDS HIV (ref=no)** |  | 0.90 | | (0,70 - 1,15) | | 0.25 |  |
| **Hospital stay characteristics** |  |  | |  | |  |  |
| **Hospital readmission (ref=no hospital readmission)** | Other acute care | 1.12 | | (1,08 - 1,16) | | <.0001 |  |
|  | Recurrent sepsis | 1.93 | | (1,85 - 2,01) | | <.0001 |  |
| **ICU admission and septic shock (ref=absence of both)** | No ICU admission and septic shock | 1.58 | | (1,46 - 1,71) | | <.0001 |  |
|  | ICU admission and septic shock | 0.84 | | (0,80 - 0,88) | | <.0001 |  |
|  | ICU admission and no septic shock | 0.75 | | (0,73 - 0,78) | | <.0001 |  |
| **Infection characteristics** |  |  | |  | |  |  |
| **Site (ref=Urinary and genital tracts)** | Gastrointestinal and abdomen | 1.00 | | (0,94 - 1,07) | | 0.88 |  |
|  | Primary bacteraemia | 1.34 | | (1,28 - 1,40) | | <.0001 |  |
|  | Bones and joints | 0.88 | | (0,79 - 0,99) | | 0.01 |  |
|  | Heart and mediastinum | 1.33 | | (1,25 - 1,42) | | <.0001 |  |
|  | Multiple | 1.34 | | (1,28 - 1,39) | | <.0001 |  |
|  | Material device | 1.20 | | (1,11 - 1,29) | | <.0001 |  |
|  | Lower respiratory tract | 1.31 | | (1,25 - 1,37) | | <.0001 |  |
|  | Skin and soft tissues | 1.59 | | (1,50 - 1,69) | | <.0001 |  |
|  | Others | 0.94 | | (0,83 - 1,07) | | 0.22 |  |

**eFigure 1.** 1-year Kaplan-Meier crude survival estimates for sepsis survivors (>15yo) according to 1-year hospital readmission for recurrent sepsis

**
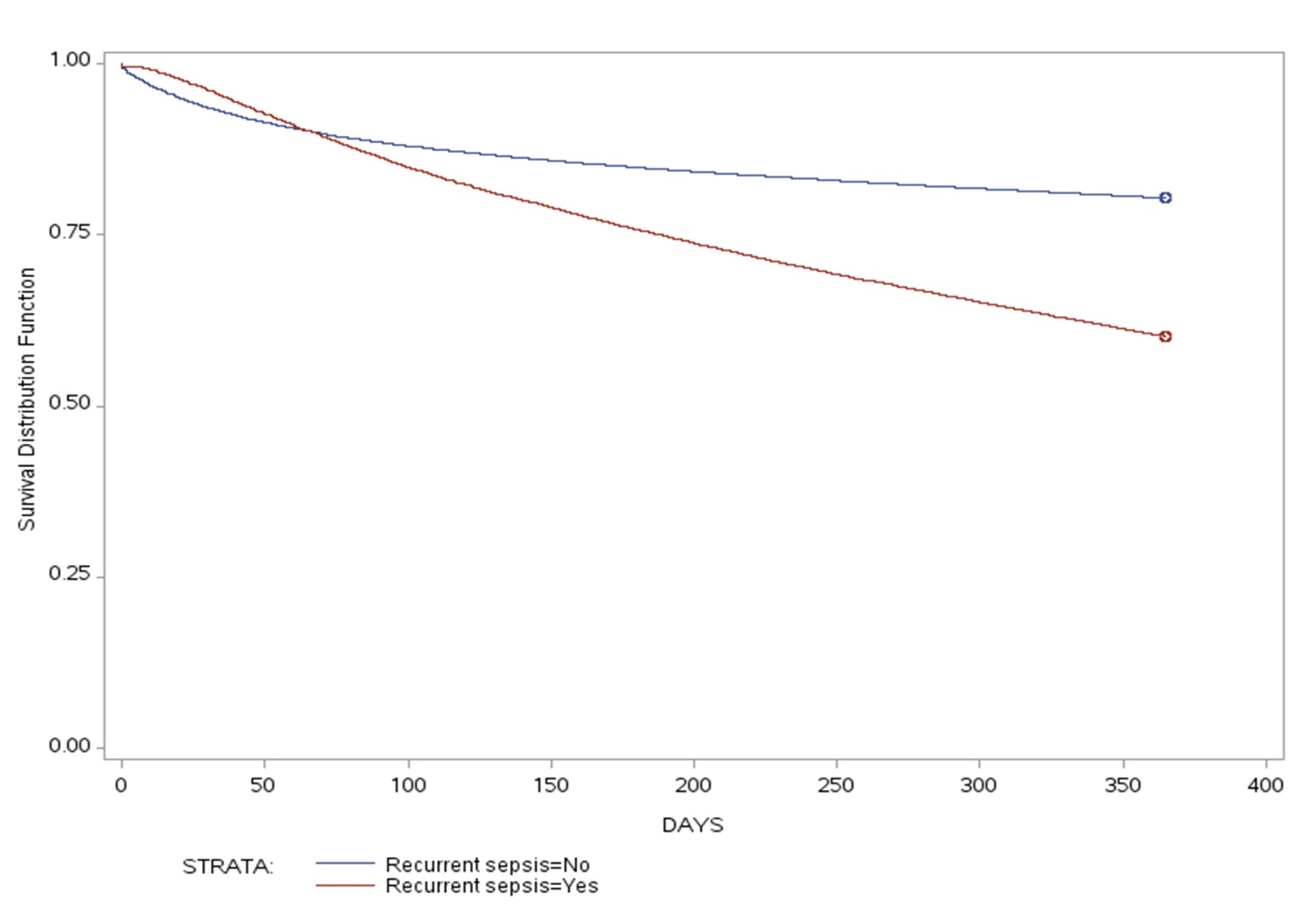
**

**References**

1. Singer M, Deutschman CS, Seymour CW, et al. The Third International Consensus Definitions for Sepsis and Septic Shock (Sepsis-3). *JAMA*. 2016;315(8):801-810. doi:10.1001/jama.2016.0287

2. Quan H, Li B, Couris CM, et al. Updating and validating the Charlson comorbidity index and score for risk adjustment in hospital discharge abstracts using data from 6 countries. *Am J Epidemiol*. 2011;173(6):676-682. doi:10.1093/aje/kwq433

3. Pandolfi F, Guillemot D, Watier L, Brun-Buisson C. Trends in bacterial sepsis incidence and mortality in France between 2015 and 2019 based on National Health Data System (Système National des données de Santé (SNDS)): a retrospective observational study. *BMJ Open*. 2022;12(5):e058205. doi:10.1136/bmjopen-2021-058205
